# From the archives: On abiotic stress signaling: An ON/OFF switch for the heat stress response in wheat, connecting gibberellin signaling and salt stress, and calcium homeostasis and stress sensitivity

**DOI:** 10.1093/plcell/koae258

**Published:** 2024-09-20

**Authors:** Leiyun Yang

**Affiliations:** Assistant Features Editor, The Plant Cell, American Society of Plant Biologists; Department of Plant Pathology, College of Plant Protection, Nanjing Agricultural University, Key Laboratory of Integrated Management of Crop Diseases and Pests, Ministry of Education, Nanjing, 210095, China; The Key Laboratory of Plant Immunity, Nanjing Agricultural University, Nanjing, 210095, China

## 2023: SUMOylation of HsfA1 regulates the heat stress response in wheat

Plants are constantly challenged by a wide range of environmental stresses, including extreme temperatures, drought, and salinity. These abiotic stresses can impair plant growth and development, leading to reduced crop yields, a problem exacerbated by climate change. Therefore, understanding the molecular mechanism underlying plant responses to abiotic stresses is crucial for sustainable agriculture. To withstand these stresses, plants have evolved complex and interconnected regulatory pathways that enable them to respond and adapt efficiently. Heat shock transcription factors (Hsfs) play a pivotal role in plant heat stress responses (HSR). Hsfs perceive heat stress (HS) and rapidly initiate transcriptional reprogramming of heat shock–responsive genes to activate HSR (Scharf et al. 2012). **Wang et al. (2023)** defined SUMOylation of HsfA1 as a highly dynamic ON/OFF molecular switch for controlling wheat HSR. After HS treatment, the *Tahsfa1* mutant exhibited reduced heat tolerance, accompanied by reduced induction of HS-responsive genes, compared with the wild type. A yeast 2-hybrid assay revealed a SUMO (small ubiquitin-related modifier) protein TaSUMO1 as a TaHsfA1 interactor. SUMO proteins can be attached to and alter the properties of substrate proteins, an important post-translational modification process called SUMOylation (Vertegaal 2022). The authors found SUMOylation of TaHsfA1 to be positively correlated with transcriptional activation activity of TaHsfA1. In vitro SUMOylation assays showed that TaHsfA1 SUMOylation is induced upon 16 °C treatment, whereas it is gradually decreased by 50 °C treatment for 3 h, suggesting that SUMOylation of TaHsfA1 is thermo-sensitive and repressed by high temperature. Further study showed that TaHsfA1 is SUMOylated predominantly on Lys459. The K459R mutation (lysine substituted by arginine) drastically reduced the SUMOylation of TaHsfA1 and abolished the transcriptional activation of TaHsfA1. This study showcases the SUMOylation of TaHsfA1 as an ON/OFF molecular switch of wheat HSR.

## 2019: Dual function of PKG in gibberellin signaling and salt stress

Moving from HS to salt stress, **Shen et al. (2019)** reported the phosphorylation and dephosphorylation activity of cGMP-dependent protein kinase (PKG) as a molecular switch for controlling gibberellin (GA) signaling and salt stress in rice. In mammalian cells, PKG perceives cyclic GMP (cGMP), an important secondary messenger, to regulate diverse cellular processes (Francis et al. 2010). Through sequence similarity searching, Shen et al. identified a rice PKG that contains a unique type 2C protein phosphatase (PP2C) domain for dephosphorylation in addition to the conserved kinase domain for phosphorylation. In vitro enzymatic assays showed that cGMP promotes the kinase activity but inhibits the phosphatase activity of PKG. A yeast 2-hybrid assay identified 22 PKG interactors, including GAMYB, a transcription factor in GA signaling. Both the *pkg* and *gamyb* mutant exhibited typical GA signaling deficiency phenotypes, such as impaired seed germination, shortened internode, and reduced pollen viability. Upon sensing cGMP, PKG interacts with and phosphorylates GAMYB in the cytoplasm, and phosphorylated GAMBY is subsequently translocated into the nucleus for transcriptional activation of GA-responsive genes. When cellular cGMP is low, the kinase activity of PKG is reduced, whereas its phosphatase activity is enhanced, resulting in de-phosphorylation of GAMYB and reduced expression of GA-responsive genes. This study presents PKG phosphorylation and de-phosphorylation activity as a dynamic switch in controlling GA signaling. Of note, many other PKG interactors are involved in osmotic stress, including salt stress responses that were reported to induce cGMP levels (Donaldson et al. 2004). Consistently, the *pgk* mutant exhibited increased sensitivity to salt stress (see Figure). These results revealed cGMP-PKG as a master regulatory cascade in response to diverse environmental stimuli.

**Figure.**
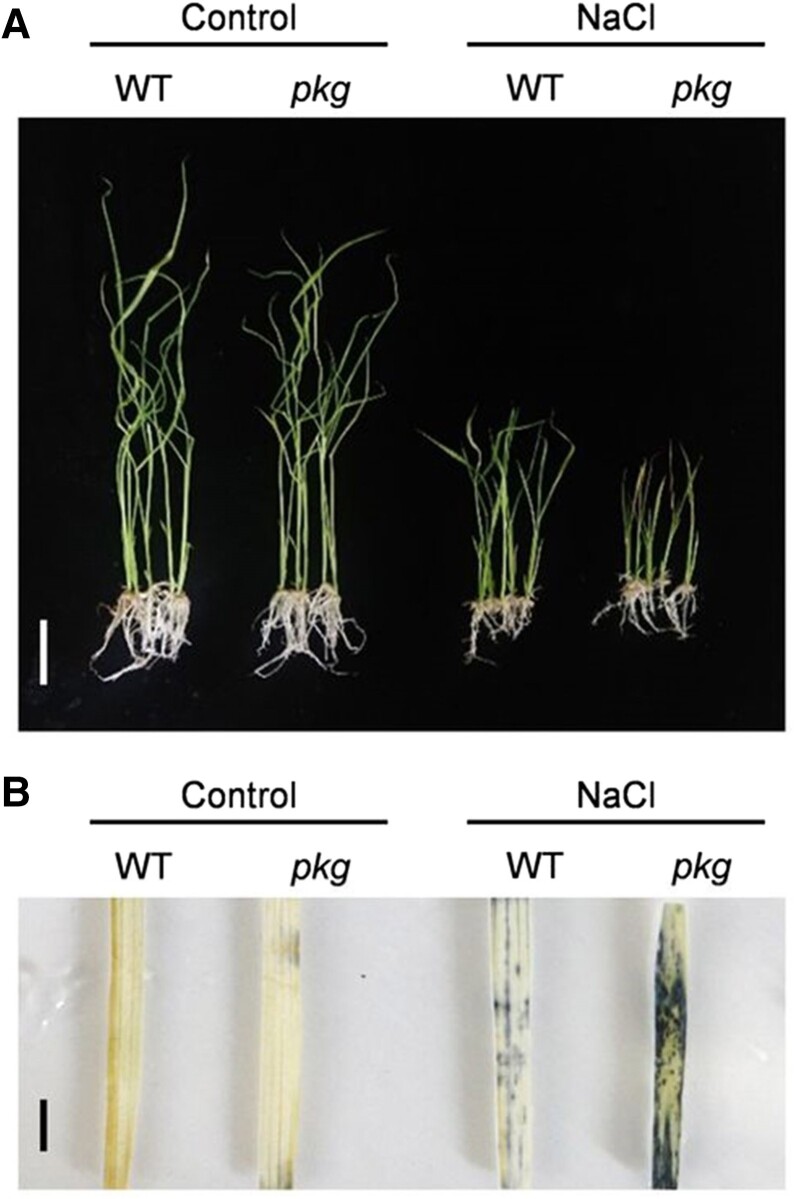
The *pkg* mutant, disrupted in GA signaling, shows increased sensitivity to salt stress. **A)** Rice growth phenotype of *pkg* mutant and WT under control and 125 mm NaCl treatment for 14 d. **B)** ROS accumulation indicated by NBT staining in the *pkg* mutant and WT leaves with and without NaCl treatment. Bars = 5 cm (A), 1 cm (B). WT, wild type. Adapted from Shen et al. (2019), Figure 11.

## 1999: Calcium homeostasis and stress sensitivity

In addition to cGMP, calcium is another well-established messenger. Calcium spikes, characterized by a transient increase of cytosolic calcium, can transduce a myriad of environmental stimuli into cellular responses (Tian et al. 2020). Calcium concentrations in different subcellular compartments are maintained at a proper level by multiple proteins through cytosolic calcium influx and/or efflux (Tian et al. 2020). **Hirschi (1999)** reported that perturbed cytosolic calcium homeostasis greatly impacts plant growth and stress responses. The Arabidopsis *CALCIUM EXCHANGER 1* (*CAX1*) gene encodes a vacuolar Ca^2+^/H^+^ antiporter responsible for calcium efflux from the cytosol. *CAX1* expression is moderately induced by Na^+^, Ni^+^, and osmotic stress and highly induced by Ca^2+^. Heterogeneous expression of Arabidopsis *CAX1* in tobacco results in increased Ca^2+^ accumulation in both shoot and root tissues, presumably through Ca^2+^ sequestration into the vacuole. The transgenic *CAX1* tobacco plants exhibited abnormal growth phenotypes such as necrotic lesions, chlorotic and twisted leaves, and reduced root mass, as well as increased sensitivity to high concentrations of MgCl_2_ and KCl. These *CAX1*-induced symptoms can be mitigated by exogenous supply of Ca^2+^. Additionally, the transgenic plants are more sensitive to cold stress. This study clearly linked Ca^2+^ homeostasis with stress sensitivity.

## Data Availability

None declared.

## Dive Curated Terms

The following phenotypic, genotypic, and functional terms are of significance to the work described in this paper:

CAX1 Gramene: AT2G38170
CAX1 Araport: AT2G38170

PGK Gramene: AT1G79550
PGK Araport: AT1G79550

calcium CHEBI: CHEBI:29320

calcium concentration Planteome: TO:0006047

NBT Gramene: Nitro blue tetrazolium
NBT Araport: Nitro blue tetrazolium
